# Herpetic Stromal Keratitis following Selective Laser Trabeculoplasty

**DOI:** 10.1155/2016/5768524

**Published:** 2016-02-17

**Authors:** Nisha Chadha, David A. Belyea, Sanjeev Grewal

**Affiliations:** ^1^Icahn School of Medicine at Mount Sinai, Department of Ophthalmology, New York, NY, USA; ^2^The George Washington University School of Medicine and Health Sciences, Department of Ophthalmology, Washington, DC, USA; ^3^Department of Veteran's Affairs, Loma Linda Healthcare System, Department of Ophthalmology, Loma Linda, CA, USA

## Abstract

This is a case report describing two cases of disciform corneal edema following uncomplicated selective laser trabeculoplasty (SLT) thought to be secondary to herpes simplex virus (HSV) given the presence of a dendrite, decreased corneal sensation, corneal thinning, and response to therapy with oral and topical antivirals. Corneal edema after SLT treatment has been reported before, but the etiology has been unclear. Our cases highlight HSV as a likely etiology, which may help with prevention and better management of such cases in the future.

## 1. Introduction

Since the introduction of selective laser trabeculoplasty (SLT) as an effective means of intraocular pressure (IOP) reduction by Latina and colleagues, SLT has been a popular form of treatment for eyes with glaucoma. The safety of SLT has also been well established with post-treatment complications being limited to infrequent transient IOP spikes and anterior chamber inflammation, both which can be well controlled with short term topical therapy [1, 2]. Recently, there have been a few case reports of corneal edema following uncomplicated SLT treatment. While the etiology is unclear, possible triggers that were considered included thermal damage, history of prostaglandin analogue use, and herpes simplex virus (HSV). However, the patients in these reports did not have a history of oral ulcers, decreased corneal sensitivity, or other stigmata of ocular herpes, limiting evidence to support this etiology [3–6]. We report two cases of corneal edema immediately following uncomplicated treatment with SLT which demonstrated HSV-association based on the presentation pattern of diffuse microcystic corneal edema which rapidly evolved into a disciform keratitis with a latent discrete area of corneal haze and thinning, along with decreased corneal sensitivity.

## 2. Case Reports

**Case 1 .**

**Figure 1:**
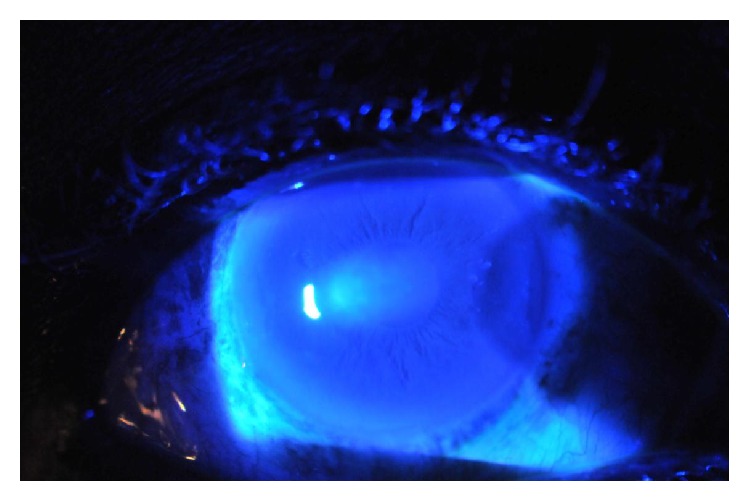
Slit lamp view under cobalt blue light of disciform keratitis and epithelitis after treatment with SLT.

A 64-year-old African American female with a history of open angle glaucoma on bimatoprost ophthalmic drops underwent uncomplicated, 360-degree SLT treatment (1.0 mJ, 134 spots) in the right eye for visual field progression. IOP before treatment was 22 mmHg and IOP 30 minutes after treatment was 21 mmHg. She was given bromfenac ophthalmic drops to use after procedure BID. She presented one day after treatment complaining of tearing and redness and was found to have a drop in visual acuity from 20/50 before treatment to 20/100. On exam she had diffuse microcystic corneal edema and Descemet's folds. Anterior chamber was deep and quiet, without cells or flare, and IOP was 15 mmHg. Reaction to bromfenac was suspected. Therefore, this drop was discontinued and the patient was started on difluprednate ophthalmic drops on which her symptoms improved. A week later, the microcystic cornea edema resolved, and a well demarcated area of circular, anterior stromal haze was present. Difluprednate ophthalmic drops were continued and on follow-up the next week, the patient's vision decreased to 20/200. On exam, in addition to the well-demarcated region of stromal haze, she had an overlying dendritic lesion that stained with fluorescein under the cobalt blue light consistent with herpetic dendrite and keratitis (Figure 1). Further testing revealed decreased corneal sensitivity. Difluprednate was discontinued and the patient was started on oral acyclovir and ganciclovir ophthalmic drops. Within two weeks, the dendrite had resolved. The patient was continued on oral acyclovir and transitioned to prednisolone acetate 1% ophthalmic drops. On this regimen, the stromal haze and vision gradually improved. Nine months later her vision was restored to her baseline of 20/50 in the setting of advanced glaucoma, and exam revealed faint residual stromal haze and thinning. Pachymetry in the affected eye had decreased from 562 microns before SLT to 508 microns after SLT. The patient also experienced a hyperopic shift from a spherical equivalent of −1.25 D preoperatively to −0.25 D postoperatively. Additionally, corneal sensation remained decreased. The patient had no previous history of herpes ophthalmicus or oral ulcers.

**Case 2 .**

**Figure 2:**
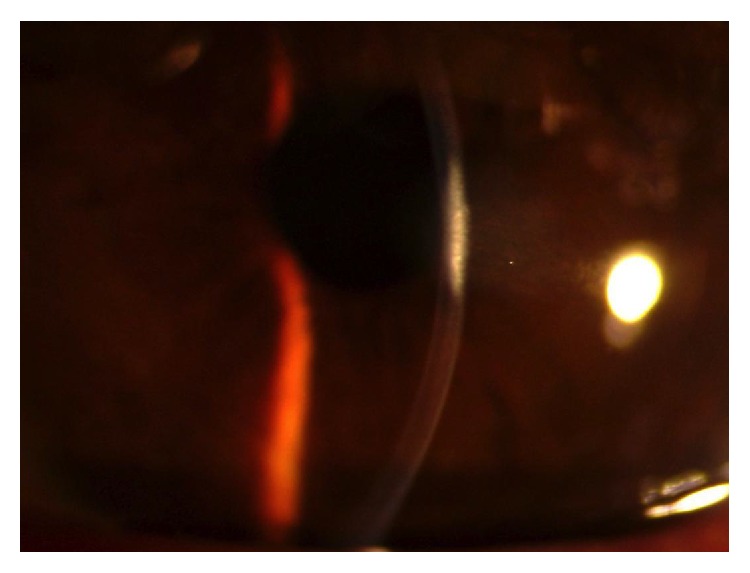
Slit beam view of stromal keratitis following SLT treatment.

A 51-year-old Lebanese female with history of open angle glaucoma on travoprost and brimonidine ophthalmic drops underwent uncomplicated, 360-degree SLT (1.0 mJ, 122 spots) in the left eye for visual field progression. IOP before treatment was 20 mmHg and 30 minutes after treatment was 21 mmHg. She was prescribed nepafenac ophthalmic drops TID after treatment. One day after treatment she complained of “hazy” vision in the treated eye. Her vision only mildly decreased from 20/25 before treatment to 20/30. On exam she was found to have diffuse microcystic corneal edema with trace Descemet's folds. Anterior chamber was deep and quiet, without cells or flare, and intraocular pressure was 19 mmHg. As reaction to nepafenac was suspected, this drop was discontinued, and the patient was started on prednisolone acetate 1% ophthalmic drops. Two days later, the microcystic corneal edema resolved but a discrete disciform region of anterior stromal haze was present paracentrally with irregular overlying epithelium (Figure 2). Herpetic keratitis was suspected and additional testing revealed decreased corneal sensation. The patient was then started on oral acyclovir, topical ganciclovir, and topical ciprofloxacin ointment. Prednisolone acetate was held until the overlying epithelium became more regular in appearance. After treatment for approximately four weeks, her anterior stromal haze resolved with only a faint scar remaining on slit lamp exam. Vision was restored to 20/20. However, the patient experienced a hyperopic shift from a spherical equivalent of −8 D before treatment to −7 D after treatment. Decreased corneal sensitivity persisted and pachymetry decreased from 533 microns before SLT to 494 microns after SLT. This patient also did not have any history of herpes ophthalmicus and denied history of cold sores.

## 3. Discussion

Our series highlights two unusual cases of diffuse corneal edema following SLT which evolved into a permanent disciform area of haze and thinning, consistent with herpetic stromal keratitis. While both patients did not have a history of ocular herpes or oral ulcers, the ubiquitous nature of HSV makes it possible. Furthermore, HSV-1 DNA has been detected in the tear film of asymptomatic individuals by PCR and postoperative HSV keratitis has been reported in patients without prior clinical history of HSV [7, 8]. Additionally, the presence of a dendritic lesion overlying the disciform keratitis in one case, decreased corneal sensitivity, thinning on pachymetry, and response to treatment with oral acyclovir and topical Zirgan suggest a herpetic etiology.

Although it is possible that the HSV could have been introduced from the lens used during treatment, this possibility is unlikely as the lens was cleaned prior to use for each patient. Additionally, in one case, other patients were treated the same day without such complications. Another possibility is that post-procedure treatment with topical steroids or NSAIDs may have triggered an episode of herpetic keratitis. However, we would expect a greater incidence of this complication following ophthalmic laser treatments given that we routinely prescribe these drops after procedure. Prostaglandin analogues, which both patients were using, could have also been responsible for these findings. However, the temporal association with SLT makes this etiology less likely. Furthermore, prostaglandin analogue associated HSV keratitis has been reported to cause dendritic herpetic keratitis but not stromal keratitis, which both of our patients developed [9, 10]. Our experience with these two cases may shed some light on the etiology of corneal edema after SLT reported in other cases.

While reactivation of herpes following intraocular surgery can occur and prophylaxis is often prescribed preoperatively, the activation or reactivation of herpes following laser procedures is less well established. There have been a few reports of herpetic keratitis following use of the excimer laser, one report following use of argon laser for peripheral iridotomy, and one report following argon laser trabeculoplasty [11–14]. These findings suggest that perhaps a more detailed history is warranted prior to initiating ophthalmic laser treatment of any kind on patients. Furthermore, if history of ocular herpes is elicited, prophylaxis should be considered and appropriate counseling delivered. Additionally, ocular herpes should be considered on the differential diagnosis of atypical ocular inflammation following selective laser trabeculoplasty and other ophthalmic laser treatments. Higher powered studies are needed to establish risk factors for corneal edema and stromal keratitis following selective laser trabeculoplasty and other ophthalmic laser treatments.
